# Diffusion MRI is superior to quantitative T2-FLAIR mismatch in predicting molecular subtypes of human non-enhancing gliomas

**DOI:** 10.1007/s00234-024-03475-z

**Published:** 2024-10-08

**Authors:** Nicholas S. Cho, Francesco Sanvito, Viên Lam Le, Sonoko Oshima, Ashley Teraishi, Jingwen Yao, Donatello Telesca, Catalina Raymond, Whitney B. Pope, Phioanh L. Nghiemphu, Albert Lai, Noriko Salamon, Timothy F. Cloughesy, Benjamin M. Ellingson

**Affiliations:** 1 Department of Radiological Sciences, David Geffen School of Medicine, University of California, Los Angeles, Los Angeles, CA USA; 2 UCLA Brain Tumor Imaging Laboratory (BTIL), Center for Computer Vision and Imaging Biomarkers, University of California, Los Angeles, Los Angeles, CA USA; 3 Department of Bioengineering, Henry Samueli School of Engineering and Applied Science, University of California, Los Angeles, Los Angeles, CA USA; 4 Medical Scientist Training Program, David Geffen School of Medicine, University of California, Los Angeles, Los Angeles, CA USA; 5 Department of Biostatistics, Fielding School of Public Health, University of California Los Angeles, Los Angeles, CA USA; 6 UCLA Neuro-Oncology Program, David Geffen School of Medicine, University of California, Los Angeles, Los Angeles, CA USA; 7 Department of Neurology, David Geffen School of Medicine, University of California, Los Angeles, Los Angeles, CA USA; 8 Department of Neurosurgery, David Geffen School of Medicine, University of California, Los Angeles, Los Angeles, CA USA; 9 Department of Psychiatry and Biobehavioral Sciences, David Geffen School of Medicine, University of California, Los Angeles, Los Angeles, CA USA

**Keywords:** T2-FLAIR mismatch sign, IDH-mutant glioma, MRI, Diffusion MRI, Digital subtraction

## Abstract

**Purpose:**

This study compared the classification performance of normalized apparent diffusion coefficient (nADC) with percentage T2-FLAIR mismatch-volume (%T2FM-volume) for differentiating between IDH-mutant astrocytoma (IDHm-A) and other glioma molecular subtypes.

**Methods:**

A total of 105 non-enhancing gliomas were studied. T2-FLAIR digital subtraction maps were used to identify T2FM and T2-FLAIR non-mismatch (T2FNM) subregions within tumor volumes of interest (VOIs). Median nADC from the whole tumor, T2FM, and T2NFM subregions and %T2FM-volume were obtained. IDHm-A classification analyses using receiver-operating characteristic curves and multiple logistic regression were performed in addition to exploratory survival analyses.

**Results:**

T2FM subregions had significantly higher nADC than T2FNM subregions within IDHm-A with ≥ 25% T2FM-volume (*P* < 0.0001). IDHm-A with ≥ 25% T2FM-volume demonstrated significantly higher whole tumor nADC compared to IDHm-A with < 25% T2FM-volume (*P* < 0.0001), and both IDHm-A subgroups demonstrated significantly higher nADC compared to IDH-mutant oligodendroglioma and IDH-wild-type gliomas (*P* < 0.05). For classification of IDHm-A vs. other gliomas, the area under curve (AUC) of nADC was significantly greater compared to the AUC of %T2FM-volume (*P* = 0.01, nADC AUC = 0.848, %T2FM-volume AUC = 0.714) along with greater sensitivity. In exploratory survival analyses within IDHm-A, %T2FM-volume was not associated with overall survival (*P* = 0.2), but there were non-significant trends for nADC (*P* = 0.07) and tumor volume (*P* = 0.051).

**Conclusion:**

T2-FLAIR subtraction maps are useful for characterizing IDHm-A imaging characteristics. nADC outperforms %T2FM-volume for classifying IDHm-A amongst non-enhancing gliomas with preserved high specificity and increased sensitivity, which may be related to inherent diffusivity differences regardless of T2FM. In line with previous findings on visual T2FM-sign, quantitative %T2FM-volume may not be prognostic.

**Supplementary Information:**

The online version contains supplementary material available at 10.1007/s00234-024-03475-z.

## Introduction

The “T2-FLAIR mismatch sign” (T2FM-sign) on T2-weighted MRI and T2-weighted FLAIR MRI is an established qualitative imaging feature with near 100% specificity for classifying isocitrate dehydrogenase-mutant 1p/19q-intact astrocytomas (IDHm-A) from IDH-mutant 1p/19-codeleted oligodendrogliomas (IDHm-O) and IDH-wild-type (IDHwt) gliomas [1–9]. Non-invasive identification of IDHm-A—and perhaps more importantly, ruling out presence of aggressive IDHwt gliomas—can be beneficial in the up-front, treatment-naïve setting to guide treatment planning discussions in the relatively younger patient populations affected by IDHm-A compared to IDHwt gliomas [10], particularly with the recent advent of mutant IDH inhibitor targeted therapies [11].

Patel et al. first defined the T2FM-sign in IDHm-A as “presence or absence of complete/near-complete hyperintense signal on T2WI, and relatively hypointense signal on FLAIR except for a hyperintense peripheral rim” [1]. However, a limitation of the T2FM-sign has remained its low *sensitivity* for identifying IDHm-A. The seminal paper by Patel et al. observed sensitivities of 22.0 and 45.5% in two cohorts and a specificity of 100% in both cohorts [1], and low sensitivities have consistently been reported in subsequent studies [2, 7, 9]. There have been several approaches to potentially increase the sensitivity of the T2FM-sign. One approach has been to utilize looser definitions of the T2FM-sign, such as a visually-estimated tumor ≥ 25% T2FM-volume threshold proposed by Lasocki et al. [12] or assessing only for T2-weighted FLAIR hyperintense rim & hypointense core and not using the T2-weighted MRI scan proposed by Li et al. [13], which achieved 100% specificity for IDHm-A with 63% and 71.3% sensitivity in their cohorts, respectively. Another approach has been to combine the T2FM-sign with quantitative MRI measures that have been previously well-described to classify IDHm-A from other molecular subgroups, such as combining T2FM-sign with apparent diffusion coefficient (ADC) from diffusion MRI [14] or normalized relative cerebral blood volume (nrCBV) from dynamic susceptibility contrast perfusion MRI [14]. However, one factor to consider about these prior approaches is that they were confined to the field’s *qualitative*, binarized (yes/no) assessment of T2FM-sign.

Recently, a *quantitative*, continuous assessment of T2FM volumetry using voxel-wise digital subtraction maps of T2-weighted and T2-weighed FLAIR MRI was introduced [9]. Cho et al. observed that quantitative ≥ 42% T2FM-volume achieved 100% specificity and 23.1% sensitivity for IDHm-A while ≥ 25% T2FM-volume still achieved high specificity of 95% with 41.5% sensitivity for IDHm-A [9], which quantitatively validated prior qualitative results by Lasocki et al. [12] who proposed a threshold of ≥ 25% T2FM-volume on visual evaluation for classifying IDHm-A.

Thus, there remains a present need for comparing and incorporating quantitative tumor %T2FM-volume with other well-known, quantitative imaging biomarkers for IDHm-A. For example, ADC values from diffusion MRI are known to be higher in IDHm-A compared to IDHm-O and IDHwt [15, 16]. ADC values are inversely related to cellular density [17], and a prior study using *qualitatively*-defined T2FM and T2-FLAIR non-mismatch (T2FNM) subregions observed that the T2FM-core subregion has higher ADC values than the T2FNM-rim subregion [18]. Given this finding, there remains a contemporary need of re-assessing previously established ADC group differences between IDHm-A and other molecular subtypes in the context of “mismatched” and “non-mismatched” IDHm-A as well as exploring subregional differences in “mismatched” IDHm-A.

The purpose of the present study was to utilize T2-FLAIR subtraction and normalized ADC (nADC) maps to characterize IDHm-A and IDHm-A subregions and to compare the classification performance of %T2FM-volume and nADC for differentiating between IDHm-A and other molecular subtypes. We hypothesized that: (**i**) T2-FLAIR subtraction map-defined T2FM subregions would have higher nADC compared to T2FNM subregions in IDHm-A with ≥ 25% T2FM-volume (“mismatched” according to Cho et al. [9]. and Lasocki et al. [12]), (**ii**) IDHm-A with ≥ 25% T2FM-volume (“mismatched”) would have higher nADC compared to IDHm-A with < 25% T2FM-volume (“non-mismatched”), and (**iii**) nADC would outperform %T2FM-volume in classifying IDHm-A from IDHm-O and IDHwt gliomas due to the inherently low sensitivity of T2FM-sign. In exploratory analyses, we also theorized that (**iv**) %T2FM-volume would not be associated with survival, as was demonstrated in prior studies using the visual T2FM-sign [1, 4, 8].

## Materials and methods

### Patient cohort

A total of 645 patients with biopsy-proven gliomas across The Cancer Imaging Archive University of California San Francisco (TCIA UCSF) [19] and our institution were initially screened. Patients with the following inclusion criteria were studied: (1) non-enhancing, adult-type diffuse glioma as classified by the World Health Organization 2021 criteria [20] (excluded *n* = 531), (2) supratentorial (excluded *n* = 2), (3) treatment-naïve except for biopsy (excluded *n* = 7), and (4) molecular status available (IDH status for all lesions and 1p/19q status if IDH-mutant; excluded *n* = 1). As a result, a total of 104 patients with 105 lesions were included in the study. This patient cohort was assessed in a prior study [9]. IDH and 1p/19q molecular status were determined by targeted next-generation sequencing, polymerase chain reaction sequencing, or immunohistochemistry and fluorescence in situ hybridization, respectively [21, 22]. Patient clinical data are summarized in Table 1.

**Table 1:** Patient characteristics

Characteristic	Patient Cohort (*n* = 104 patients with *n* = 105 lesions)
Age: Mean (Range)	42 (22–79)
Sex: M/F	59/45
Diagnosis: n (%)	
IDHm Astrocytoma Grade 2 Grade 3 Grade 4 IDHm Oligodendroglioma Grade 2 Grade 3 IDHwt Glioma	65 (61.9%) 44 20 1 18 (17.1%) 17 1 22 (21.0%)

IDHm = isocitrate dehydrogenase mutant; IDHwt = isocitrate dehydrogenase wild-type

### Image acquisition and pre-processing

All patients underwent T2-weighted, T2-weighted FLAIR, and diffusion MRI on 3T scanners. T2-weighted and T2-weighted FLAIR were obtained using previously described protocols [9, 19]. ADC maps were generated from diffusion MRI datasets acquired with b-values of 0 and 1000 s/mm^2^ (see Supplementary Table 1 for diffusion MRI protocol information). The TCIA data [19] were already pre-processed and registered to the 3D T2-weighted FLAIR MRI (Advanced Normalization Tools) and skull-stripped using “brain_mask” (https://www.github.com/ecalabr/brain_mask/), and the institutional data were pre-processed using an analogous pipeline of registering to the 3D T1-post-contrast MRI (*tkregister2*; Freesurfer [23]| *flirt*: Functional Magnetic Resonance Imaging of the Brain Software Library [24]) and skull-stripped using “HD-BET” (https://github.com/MIC-DKFZ/HD-BET) [25]. Normalized ADC (nADC) maps were created by voxel-wise dividing ADC by the mean ADC value of 3 spherical VOIs in the normal appearing white matter (NAWM) of the contralateral centrum semiovale [26].

### T2-FLAIR subtraction maps

Voxel-wise T2-FLAIR digital subtraction maps were generated for each patient as previously described [9]. In brief, an additional, refined co-registration of the skull-stripped T2-weighted and T2-weighted FLAIR MRI was performed using FLIRT. Then, images were z-score- and NAWM-normalized to the contralateral centrum semiovale so that the NAWM signal intensity would be ~ 0. Lastly, the normalized T2-weighted and T2-weighted FLAIR MRI were voxel-wise subtracted to create T2-FLAIR subtraction maps. A consistent threshold of 0 on the T2-FLAIR subtraction maps was used for all analyses, where positive values corresponded to T2FM-subregions and negative values corresponded to T2FNM-subregions within the tumor.

### Brain tumor imaging analysis

All initial tumor VOI segmentations for the institutional data were performed by a lab member with 2 years of experience in tumor segmentation analysis (N.S.C.). The institutional and provided TCIA tumor segmentations were further refined via a semi-automated thresholding method using Analysis of Functional NeuroImages (AFNI) software (https://afni.nimh.nih.gov) [27] for consistency. Macroscopic cysts and CSF were excluded from the tumor segmentations. Lastly, a radiologist with 11 years of experience in neuroimaging analysis (S.O.) inspected all final tumor VOI segmentations while being blinded to the clinical data. T2FM and T2FNM subregion VOIs were then created from the tumor VOIs using the T2-FLAIR subtraction maps, and T2FM and T2FNM volumes were calculated to quantify percentage T2FM-volume (%T2FM-volume). IDHm-A were stratified based on ≥ 25% T2FM-volume (“mismatched” IDHm-A) or < 25% T2FM-volume (“non-mismatched” IDHm-A) using previously-defined thresholds [9, 12]. Median nADC values from the tumor, T2FM subregion, and T2FNM subregion were also obtained.

### Statistical analysis

GraphPad Prism software was used for statistical analyses. Paired t-tests were performed to assess differences in nADC between T2FM and T2FNM subregions IDHm-A with ≥ 25% T2FM-volume. Repeated-measures ANOVA tests with post-hoc Holm-Sidak corrections were performed to assess group differences in nADC between different molecular types. Unpaired t-tests were performed to assess differences in nADC across IDHm-A tumor grades. Paired ROC curve analyses of %T2FM-volume and nADC to classify IDHm-A from IDHm-O/IDHwt (in line with the diagnostic usage of visual T2FM-sign [1, 12]), IDHm-A from IDHm-O, and IDHm-A from IDHwt were performed, and the DeLong test was performed to compare the paired area under curve (AUC) values of nADC versus %T2FM-volume for each classification pairing. Multiple logistic regression was performed to assess the classification performance of the combination of nADC and %T2FM-volume and the combination of nADC, %T2FM-volume, and age for classifying IDHm-A. Survival analysis was restricted to the TCIA cohort-only because all data from the institutional data were censored in terms of overall survival, and one patient with 2 lesions was excluded from survival analysis. Log-rank tests were performed assessing any relationships of nADC, %T2FM-volume, tumor volume, and molecular status as categorical variables with overall survival. Univariate and multivariate Cox survival analysis were performed on the same variables to assess any relationships with overall survival as continuous measures while controlling for factors such as age, extent of resection, and grade. Significance was set at α = 0.05 for all analyses.

## Results

**Fig. 1:**
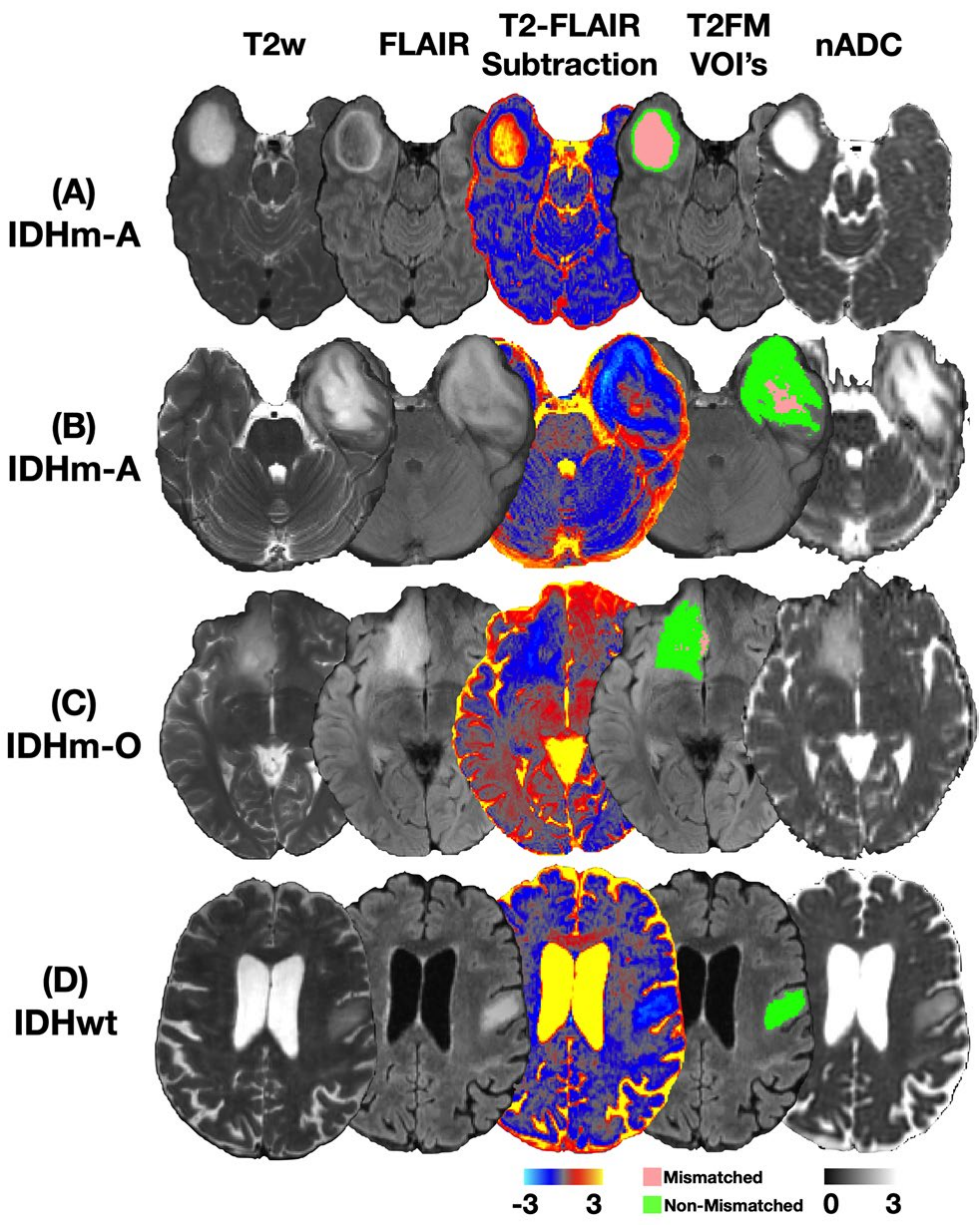
Four representative cases with quantitative T2-FLAIR subtraction and nADC maps. (**A**) Patient A is a 36-year-old male diagnosed with IDH-mutant astrocytoma (IDHm-A) with 3.02 nADC and 53.5% T2-FLAIR mismatch volume (T2FM-volume) (“mismatched”). (**B**) Patient B is a 36-year-old female diagnosed with IDHm-A with 2.15 nADC and 10.9% T2FM-volume (“non-mismatched”). (**C**) Patient C is a 27-year-old female diagnosed with IDH-mutant oligodendroglioma (IDHm-O) with 1.78 nADC and 6.8% T2FM-volume. (**D**) Patient D is a 75-year-old female diagnosed with IDH-wild type (IDHwt) glioma with 1.62 nADC and < 1% T2FM-volume. Tumor segmentation volumes of interests (VOIs) denoting T2FM subregions (pink) and T2-FLAIR non-mismatch (T2FNM) subregions (green) are shown. IDHm-A = isocitrate dehydrogenase mutant astrocytoma; IDHm-O = isocitrate dehydrogenase mutant oligodendroglioma; IDHwt = isocitrate dehydrogenase wild type glioma; T2FM = T2-FLAIR mismatch; nADC = normalized apparent diffusion coefficient

Four representative cases are shown in Fig. 1. Figure 1A shows a 36-year-old male patient who was diagnosed with an IDHm-A exhibiting a high median nADC of 3.02 and 53.5% T2FM-volume (“mismatched” IDHm-A). Figure 1B shows a 36-year-old female patient who was diagnosed with an IDHm-A exhibiting a moderately high median nADC of 2.15 nADC with only 10.9% T2FM-volume (“non-mismatched” IDHm-A). Figure 1C shows a 27-year-old female patient who was diagnosed with an IDHm-O exhibiting a lower median nADC of 1.78 and 6.8% T2FM-volume. Figure 1D shows a 75-year-old female patient who was diagnosed with an IDHwt glioma exhibiting a low median nADC of 1.62 and < 1% T2FM-volume.

**Fig. 2:**
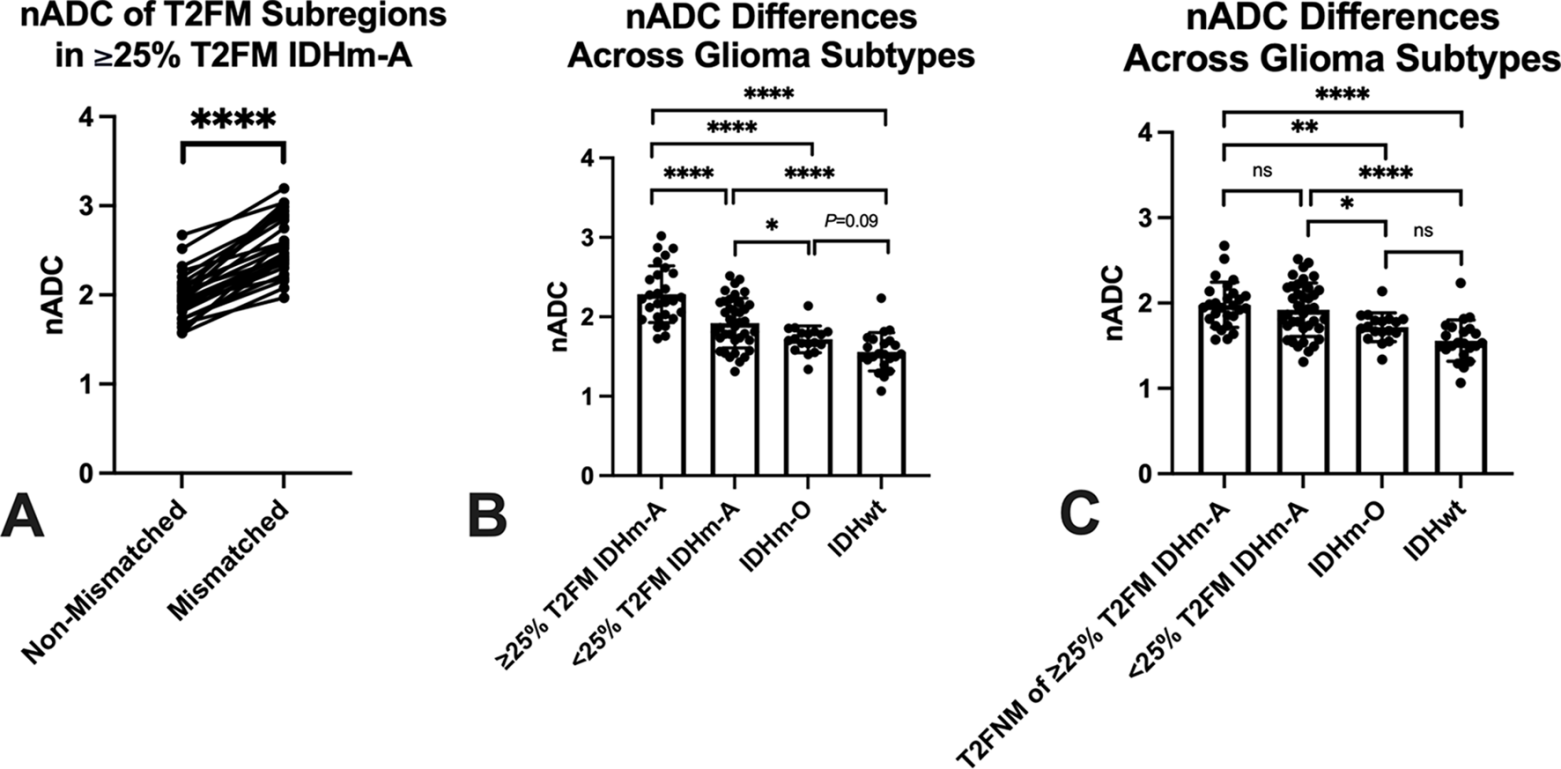
Intra-tumoral and group nADC differences based on quantitative T2-FLAIR mismatch and glioma molecular subtypes. T2-FLAIR mismatch (T2FM) subregions of IDH-mutant astrocytomas (IDHm-A) with ≥ 25% T2FM-volume had significantly higher nADC compared to T2-FLAIR non-mismatch (T2FNM) subregions (*P* < 0.0001, **A**). IDHm-A with ≥ 25% T2FM-volume had significantly higher nADC compared to IDHm-A with < 25% T2FM-volume (*P* < 0.0001, **B**) and both IDHm-A subgroups had significantly higher nADC compared to IDH-mutant oligodendroglioma (IDHm-O) (≥ 25% T2FM-volume IDHm-A: *P* < 0.0001, < 25% T2FM-volume IDHm-A: *P* = 0.03, **B**) and IDH-wild type glioma (IDHwt) (both *P* < 0.0001, **B**). T2FNM subregions of IDHm-A with ≥ 25% T2FM-volume also had significantly higher nADC compared to IDHm-O (*P* = 0.0063, **C**) and IDHwt (*P* < 0.0001, **C**), but no difference with IDHm-A with < 25% T2FM-volume (*P* = 0.39, **C**) IDHm-A = isocitrate dehydrogenase mutant astrocytoma; IDHm-O = isocitrate dehydrogenase mutant oligodendroglioma; IDHwt = isocitrate dehydrogenase wild type glioma; T2FM = T2-FLAIR mismatch; nADC = normalized apparent diffusion coefficient; ns = not significant; * denotes *P* < 0.05; ** denotes *P* < 0.01; *** denotes *P* < 0.001; **** denotes *P* < 0.0001

Within IDHm-A exhibiting ≥ 25% T2FM-volume (“mismatched”), there was significantly higher nADC in T2FM-subregions compared to T2FNM subregions (*P* < 0.0001, mean difference = 0.58, Fig. 2A). When assessing all IDHm-A, IDHm-A with ≥ 25% T2FM-volume (“mismatched”) had significantly higher whole tumor nADC compared to IDHm-A with < 25% T2FM-volume (“non-mismatched”) (*P* < 0.0001, Fig. 2B). Across glioma molecular subtypes while considering IDHm-A with ≥ 25% T2FM-volume and IDHm-A with < 25% T2FM-volume as separate entities, both IDHm-A subgroups demonstrated significantly higher nADC compared to IDHm-O (*P* < 0.0001 for ≥ 25% T2FM-volume IDHm-A, *P* = 0.03 for < 25% T2FM-volume IDHm-A, Fig. 2B) and IDHwt (*P* < 0.0001 for both ≥ 25% and < 25% T2FM-volume IDHm-A, Fig. 2B). There was also a trend towards significance for increased nADC in IDHm-O compared to IDHwt after multiple comparisons p-value correction (*P* = 0.09, Fig. 2B). The T2FNM-subregion of IDHm-A with ≥ 25% T2FM-volume demonstrated no significant difference in nADC with the whole tumor nADC of IDHm-A with < 25% T2FM-volume (*P* = 0.39, Fig. 2C). T2FNM-subregions of IDHm-A with ≥ 25% T2FM-volume still demonstrated significantly higher nADC compared to IDHm-O (*P* = 0.0063, Fig. 2C) and compared to IDHwt (*P* < 0.0001, Fig. 2C). There were no significant differences in nADC between grade 2 and grade 3 IDHm-A, whether across all IDHm-A (*P* = 0.56), only IDHm-A ≥ 25% T2FM-volume (*P* = 0.38), or only IDHm-A < 25% T2FM-volume (*P* = 0.22).

**Fig. 3:**
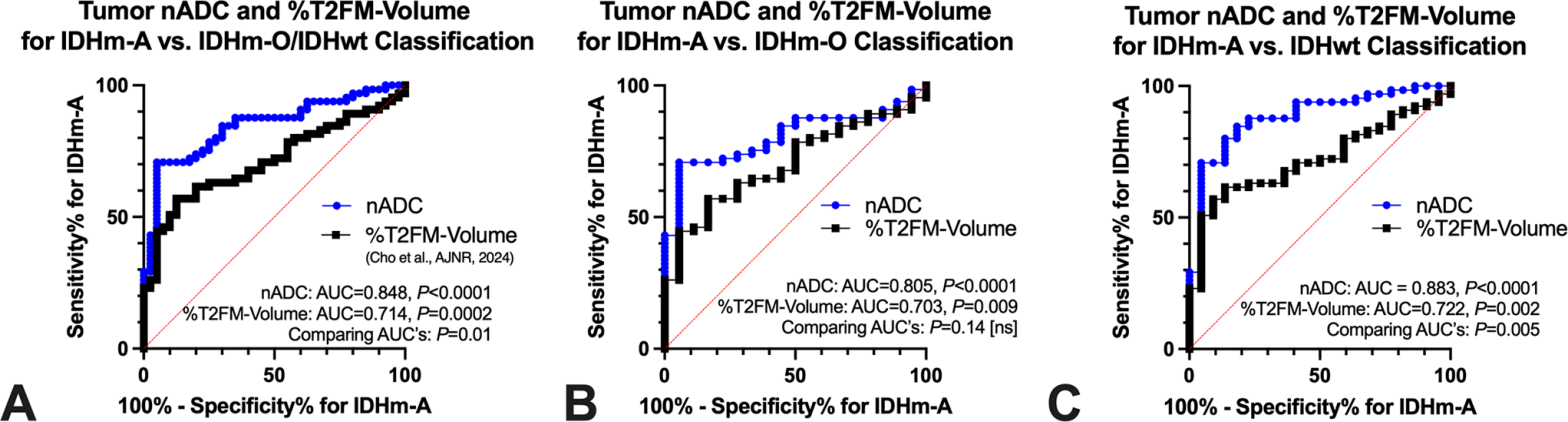
Comparing diagnostic performance of tumor nADC and percentage T2-FLAIR mismatch volume for IDH-mutant astrocytoma classification. The area under curve (AUC) of nADC was significantly higher than the AUC of percentage T2-FLAIR mismatch volume (%T2FM-volume) for classifying IDH-mutant astrocytoma (IDHm-A) from IDH-mutant oligodendroglioma (IDHm-O) and IDH-wild type (IDHwt) (*P* = 0.01 comparing AUC’s, **A**). The AUC of nADC was higher, but not significantly, than the AUC of %T2FM-volume for classifying IDHm-A from IDHm-O (*P* = 0.14 comparing AUC’s, **B**) and significantly higher for classifying IDHm-A from IDHwt (*P* = 0.005 comparing AUC’s, **C**). Refer to Table 2 for summary cutoffs of %T2FM-volume and nADC for molecular classification IDHm-A = isocitrate dehydrogenase mutant astrocytoma; IDHm-O = isocitrate dehydrogenase mutant oligodendroglioma; IDHwt = isocitrate dehydrogenase wild type glioma; T2FM = T2-FLAIR mismatch; nADC = normalized apparent diffusion coefficient; ns = not significant

Paired ROC analyses were performed to compare the diagnostic performance of nADC vs. %T2FM-volume in differentiating IDHm-A from other molecular types. Both nADC and %T2FM-volume classified IDHm-A vs. IDHm-O/IDHwt individually (*P* < 0.0001 and *P* = 0.0002, respectively), but the AUC of nADC was significantly greater than the AUC of %T2FM-volume (nADC AUC = 0.848, %T2FM-volume AUC = 0.714 [9], *P* = 0.01 comparing AUC’s, Fig. 3A). In post-hoc analyses, the AUC of nADC remained greater than the AUC of %T2FM-volume for classifying IDHm-A just from IDHm-O (Fig. 3B) and IDHm-A just from IDHwt (Fig. 3C), although the AUC difference was significant only for classification from IDHwt (*IDHm-A vs. IDHm-O*: nADC AUC = 0.805, %T2FM-volume AUC = 0.703, *P* = 0.14 comparing AUC’s| *IDHm-A vs. IDHwt*: nADC AUC = 0.883, %T2FM-volume AUC = 0.722, *P* = 0.005 comparing AUC’s). Table 2 summarizes empiric thresholds of nADC and %T2FM-volume for achieving 100% and ~ 95% specificity for the ROC analyses in Fig. 3, and the results show that nADC has greater sensitivity for IDHm-A compared to %T2FM-volume at these high-specificity thresholds (e.g. *IDHm-A vs. IDHm-O/IDHwt* with 95% specificity: nADC = 70.8% sensitivity, %T2FM-volume = 41.5% sensitivity). Multiple logistic regression results combining (**i**) nADC and %T2FM-volume and (**ii**) nADC, %T2FM-volume, and age demonstrated only *marginal* increases in AUC for classifying IDHm-A compared to using nADC-alone (*IDHm-A vs. IDHm-O/wt*: AUC 0.848 to 0.880, *IDHm-A vs. IDHm-O*: AUC 0.805 to 0.816, *IDHm-A vs. IDHwt*: AUC 0.883 to 0.938) (Supplementary Table 2).

**Table 2:** Summary of nADC and %T2FM-Volume thresholds for classifying IDH-mutant astrocytomas with 100% specificity and ~ 95% specificity

Classification: IDHm-A vs. IDHm-O/IDHwt		
Threshold	Sensitivity (IDHm-A)	Specificity (IDHm-A)
nADC > 1.864	70.8%	95.0%
%T2FM-Volume > 25.00%^+^	41.5%	95.0%
nADC > 2.240	29.2%	100%
%T2FM-Volume > 42.00%^+^	23.1%	100%
**Classification: IDHm-A vs. IDHm-O**		
**Threshold**	**Sensitivity (IDHm-A)**	**Specificity (IDHm-A)**
nADC > 1.864	70.8%	94.4%
%T2FM-Volume > 22.05%	44.6%	94.4%
nADC > 2.145	43.1%	100%
%T2FM-Volume > 37.42%	26.2%	100%
**Classification: IDHm-A vs. IDHwt**		
**Threshold**	**Sensitivity (IDHm-A)**	**Specificity (IDHm-A)**
nADC > 1.849	70.8%	95.5%
%T2FM-Volume > 13.94%	50.8%	95.5%
nADC > 2.240	29.2%	100%
%T2FM-Volume > 41.77%	23.1%	100%

^+^ Previously reported in Cho et al., AJNR, 2024; IDHm-A = isocitrate dehydrogenase mutant astrocytoma; IDHm-O = isocitrate dehydrogenase mutant oligodendroglioma; IDHwt = isocitrate dehydrogenase wild type glioma; %T2FM-volume = percentage T2-FLAIR mismatch volume; nADC = normalized apparent diffusion coefficient

For exploratory survival analysis, only 4 out of 44 patients with IDHm-A (9%) reached overall survival endpoint. Log-rank tests showed no significant association between %T2FM-volume and overall survival in IDHm-A (*P* = 0.20, ≥ 2% T2FM-volume median survival = 2191 days, < 2% T2FM-volume median survival undefined, Mantel Haenszel hazard ratio = 4.76 (95% CI: 0.43–52.79), Supplementary Fig. 1A), but there were trends towards significance for nADC (*P* = 0.07 with nADC ≥ 2.07 associated with longer OS, Mantel Haenszel hazard ratio = 0.12 (95% CI: 0.01–1.20), Supplementary Fig. 1B) and tumor volume (*P* = 0.051 with volume ≥ 60mL associated with shorter OS, Mantel Haenszel hazard ratio = 16.14 (95% CI: 0.98–264.9), Supplementary Fig. 1C) despite the large proportion of censored patients. Log-rank tests showed significant differences in overall survival between IDHwt, IDHm-O, and IDHm-A with OS_IDHwt_ < OS_IDHm−A_ < OS_IDHm−O_ (*P* < 0.0001, Supplementary Fig. 1D). Cox survival analysis assessing overall survival in IDHm-A with %T2FM-volume, nADC, and tumor volume as continuous measures demonstrated no significant results in univariate analyses (*P* > 0.05) or multivariate analyses controlling for age, extent of resection, and grade (*P* > 0.05).

## Discussion

Results from the current study suggest that diffusivity alterations may be a better discriminator for IDHm-A compared with the presence of T2FM. Our results suggest the previously-described low sensitivity of T2FM-sign for IDHm-A may not have necessarily been limited due to its previously qualitative and binarized assessment [1], but instead T2FM may be a feature with an inherently low-sensitivity for IDHm-A [9]. Consistent with the previous work from Lee et al. [14] who found that the combination of ADC characteristics and *visual* T2FM-sign improved the classification performance of IDHm-A from IDHwt gliomas, the present study observed increased performance when using the combination of nADC and *quantitative* %T2FM-volume; however, the present results also suggest that diffusion MRI alone may be sufficient to identify non-enhancing IDHm-A with preserved high specificity (~ 95–100%) and improved sensitivity compared to %T2FM-volume.

In corroboration of the findings reported by Foltyn et al. [18], the current study documented a significantly higher ADC in the T2-FLAIR subtraction map-defined T2FM-core subregions in IDHm-A compared to T2FNM-rim subregions. This finding may be explained by both the higher expression of mTOR-related genes in IDHm-A exhibiting T2FM [1, 28] resulting in higher proliferation [29, 30] and correspondingly lower ADC [31] in the rim as well as the presence of increased water mobility due to microcystic changes or enlarged intercellular space within the T2FM core region [1, 5, 8, 28]. Interestingly, the higher ADC in T2FM-areas raises a possibility that prior studies that established ADC differences across gliomas—namely, highest ADC in IDHm-A, then IDHm-O, then IDHwt [15, 16]—were potentially biased by the proportion of IDHm-A exhibiting T2FM. As a result, the present study adds to the literature by re-assessing nADC glioma differences using ≥ 25% T2FM-volume IDHm-A (“mismatched) and < 25% T2FM-volume IDHm-A (“non-mismatched”) as separate tumor entities. Importantly, even IDHm-A with < 25% T2FM-volume still had higher nADC compared to IDHm-O and IDHwt, which suggests that there are diffusivity changes in IDHm-A inherent to their tumor biology that are not necessarily solely explained by the development of microcystic changes in T2FM-regions. Furthermore, these analyses demonstrate the value of T2-FLAIR subtraction maps in combination with whole tumor segmentations for characterizing “mismatched” IDHm-A and their tumor subregions via volumetrics and quantitative image feature extraction.

Exploratory analysis found no significant association between quantitative %T2FM-volume within IDHm-A and survival, which appears in line with previous studies [1, 4, 8]. Similarly, lower nADC and larger tumor volume trended towards lower overall survival in IDHm-A, which is also consistent with prior studies [32–34]. However, it should be noted that our findings warrant cautious interpretation given that only 9% of the analyzed IDHm-A patients died during the observation period. While the median overall survival of the analyzed IDHm-A was still a considerable ~ 3 years including censored data, the median overall survival of low-grade IDHm-A is ~ 9 years [35], which presents a limitation of this study. Future studies with more mature survival data are necessary to confirm this observation.

### Limitations

This study has limitations that should be addressed. Further analyses on expanded and/or independent external cohorts would be valuable to validate our findings that nADC is superior to quantitative %T2FM-volume for imaging-based classification of IDHm-A and that %T2FM-volume is not prognostic within IDHm-A. Additionally, a whole tumor segmentation-based processing pipeline was chosen for this study to maximize the technical rigor for quantifying %T2FM-volume and nADC. While suitable for research settings, this processing pipeline may not be as directly translatable to clinical settings. Future studies may consider utilizing single-slice or “hot-spot” nADC analyses as described in some prior studies on gliomas [16, 36]. Nevertheless, our proposed pipeline may have future clinical applicability for the management of non-enhancing gliomas as the automated capabilities of clinical PACS systems continue to expand, including with volumetric tumor segmentations and feature extraction [37, 38].

## Conclusions

Diffusion MRI is better than %T2FM-volume for classifying IDHm-A amongst non-enhancing gliomas, and quantitative %T2FM-volume may not be prognostic in terms of predicting overall survival in non-enhancing human gliomas.

## Electronic supplementary material

Below is the link to the electronic supplementary material.

Supplementary Material 1

## Abbreviations

ADC: Apparent diffusion coefficient
AUC: Area under curve
IDH: Isocitrate dehydrogenase
IDHm-A: Isocitrate dehydrogenase mutant astrocytoma
IDHm-O: Isocitrate dehydrogenase mutant oligodendroglioma
IDHwt: Isocitrate dehydrogenase wild-type glioma
MRI: Magnetic resonance imaging
nADC: Normalized apparent diffusion coefficient
NAWM: Normal appearing white matter
ROC: Receiver-operating characteristic
T2FM: T2-FLAIR mismatch
T2FNM: T2-FLAIR non-mismatch
%T2FM-Volume: Percentage T2-FLAIR mismatch volume
TCIA: The Cancer Imaging Archive
VOI: Volume of interest
