# Supplementary material for: Diffusion MRI is superior to quantitative T2-FLAIR mismatch in predicting molecular subtypes of human non-enhancing gliomas
Source: Neuroradiology. 2024 Oct 8;66(12):2153–62. doi: 10.1007/s00234-024-03475-z (PMC11611930; doi:10.1007/s00234-024-03475-z)
Supplement: Supplementary file 1 — Supplementary Material 1 [file 234_2024_3475_MOESM1_ESM.pdf]

Diffusion MRI is superior to quantitative T2-FLAIR mismatch in predicting molecular subtypes of human non-enhancing gliomas

(Supplement submitted to Neuroradiology)

Supplementary Table 1. Diffusion MRI Protocol Information

Parameters	TCIA UCSF Subjects+	Institutional Subjects
TR (ms)	10000	4100–11500
TE (ms)	99	64-100
Slice Thickness (mm)	2	2
Matrix	256 x 256	128 x 128
FOV (cm)	23	23–25.6
b-value (s/mm^2)	1000	1000

TCIA = The Cancer Imaging Archive; UCSF = University of California, San Francisco

+Retrieved from Calabrese E, Villanueva-Meyer JE, Rudie JD, Rauschecker AM, Baid U, Bakas S, Cha S, Mongan JT, Hess CP (2022) The University of California San Francisco Preoperative Diffuse Glioma MRI Dataset. Radiol Artif Intell 4: e220058 doi:10.1148/ryai.220058

Supplementary Table 2. Summary of Multiple Logistic Regression Results of nADC, %T2FM-Volume, and Age for Classifying IDH-mutant Astrocytomas

**Classification: IDHm-A vs. IDHm-O/IDHwt**		
**Model Variables**	**Area Under Curve**	**P-value**
nADC-alone (ROC in Fig 3A)	0.848	<0.0001
nADC and %T2FM-Volume	0.851	<0.0001
nADC, %T2FM-Volume, and Age	0.880	<0.0001
**Classification: IDHm-A vs. IDHm-O**		
**Model Variables**	**Area Under Curve**	**P-value**
nADC-alone (ROC in Fig 3B)	0.805	<0.0001
nADC and %T2FM-Volume	0.810	<0.0001
nADC, %T2FM-Volume, and Age	0.816	<0.0001
**Classification: IDHm-A vs. IDHwt**		
**Model Variables**	**Area Under Curve**	**P-value**
nADC-alone (ROC in Fig 3C)	0.883	<0.0001
nADC and %T2FM-Volume	0.886	<0.0001
nADC, %T2FM-Volume, and Age	0.938	<0.0001

IDHm-A = isocitrate dehydrogenase mutant astrocytoma; IDHm-O = isocitrate dehydrogenase mutant oligodendroglioma; IDHwt = isocitrate dehydrogenase wild type glioma; %T2FM-volume = percentage T2-FLAIR mismatch volume; nADC = normalized apparent diffusion coefficient; ROC = receiver-operating characteristic

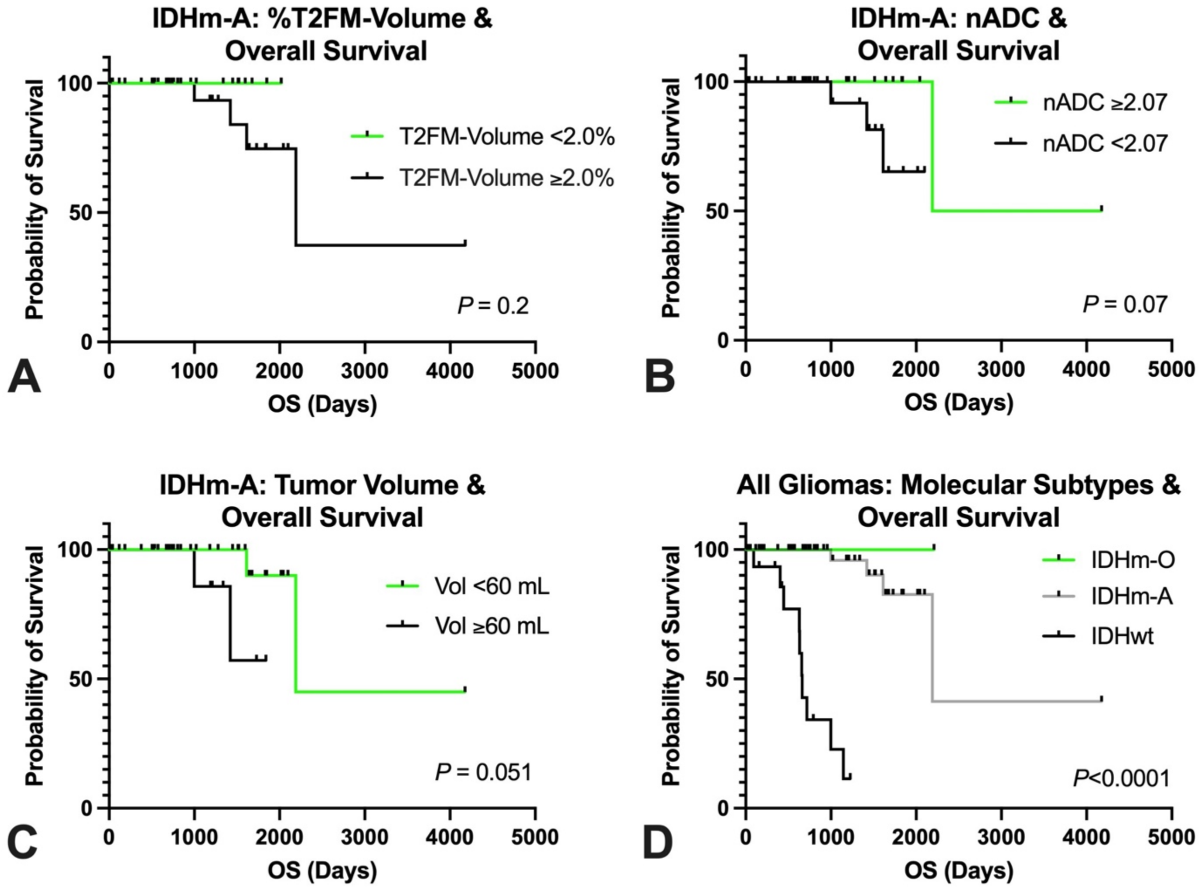

Supplementary Figure 1. Summary of Log-Rank Survival Analysis. There was no significant association of %T2FM-volume with overall survival within IDH-mutant astrocytomas (*P*=0.2, **A**), but there were trends towards significance for normalized ADC (nADC) (*P*=0.07, **B**) and tumor volume (*P*=0.051, **C**). There were significant differences in overall survival across molecular subtypes.

IDHm-A = isocitrate dehydrogenase mutant astrocytoma; IDHm-O = isocitrate dehydrogenase mutant oligodendroglioma; IDHwt = isocitrate dehydrogenase wild type glioma; %T2FM-volume = percentage T2-FLAIR mismatch volume; nADC = normalized apparent diffusion coefficient; OS = overall survival
